# DISSEMINATION OF UVEAL MELANOMA AFTER DIAGNOSTIC BIOPSY WITH 23-GAUGE VITRECTOR

**DOI:** 10.1097/IAE.0000000000004573

**Published:** 2025-07-07

**Authors:** Hung-Da Chou, Rodrigo Anguita, Caroline Thaung, Lamis Alharby, Guy S. Negretti, Lyndon da Cruz, Mandeep S. Sagoo

**Affiliations:** * Ocular Oncology Service, Moorfields Eye Hospital, London, United Kingdom;; † Chang Gung Memorial Hospital, Linkou Main Branch, Taoyuan, Taiwan and College of Medicine, Chang Gung University, Taoyuan, Taiwan;; ‡ Department of Ophthalmology, Inselspital, University Hospital of Bern, Bern, Switzerland;; § NIHR Biomedical Research Centre for Ophthalmology at Moorfields Eye Hospital and UCL Institute of Ophthalmology, London, United Kingdom; and; ¶ Vitreoretinal Service, Moorfields Eye Hospital, London, United Kingdom.

**Keywords:** biopsy, complication, melanoma, ocular oncology, pathology, proton

## Abstract

**Purpose:**

To report a case with tumor dissemination after diagnostic choroidal biopsy.

**Methods:**

Multimodal imaging and histopathology correlation.

**Results:**

A 64-year-old man presented with an atypical uveal mass and underwent a diagnostic transretinal 23-gauge vitrector biopsy to diagnose melanoma. Postoperative course was complicated by prolonged vitreous hemorrhage which required vitrectomy, delaying proton beam treatment by two months. One year after the biopsy, the tumor cells had disseminated to the retina, optic nerve, iris, iridocorneal angle, and extraocularly via the vitrectomy port. The patient died from metastatic melanoma 1 year later. To the best of our knowledge, there are 10 prior cases with biopsy-related tumor seeding in the literature, with risk features including multiple vitreous surgeries, a long interval between biopsy and treatment, and high-risk melanoma cytogenetics. Severe vitreous hemorrhage following biopsy further postpone treatments or warrant vitrectomy, both increasing the risk of tumor spread.

**Conclusion:**

Biopsy of choroidal melanoma, especially when complicated by severe vitreous hemorrhage, can lead to dissemination of the malignant tumor.

## Abstract 2

Nonclearing vitreous hemorrhage following diagnostic choroidal biopsy with vitrector may complicate the clinical course, postpone the definitive treatment, and increase the risk of melanoma dissemination.

Most uveal melanomas are diagnosed clinically without needing a biopsy. However, many patients with melanoma undergo a prognostic biopsy for cytogenetic analysis, to estimate metastatic risk and survival. Various groups have demonstrated that such prognostic biopsies have a low risk of tumor seeding, partly because definitive treatment is usually started at the time of or soon after biopsy.^1^

In contrast, some choroidal tumors that do not show typical features of melanoma still warrant a diagnostic biopsy. In these diagnostic biopsies, treatment is not always planned before the procedure and only starts after the biopsy results are received. Therefore, there is often a longer time gap between the biopsy and the definitive treatment, posing a risk of tumor seeding. In this study, we report a case with tumor dissemination after a diagnostic biopsy and discuss the considerations and management strategies.

## Methods

This is a retrospective case report with demographics and serial ophthalmic examinations, including best-corrected visual acuity, intraocular pressure, ultra-wide-field fundus photography (Optos PLC, Dunfermline, United Kingdom), high-resolution ultrasonography (ACUSON Juniper Ultrasound System; Seimens Munich, Germany), ultrasound biomicroscopy (Lumibird Medical, Cournon d’Auvergne Cedex, France), spectral-domain optical coherence tomography (Spectralis HRA-OCT system; Heidelberg Engineering, Heidelberg, Germany), and histopathology microfilms. Informed consent was obtained from the deceased patient's next of kin.

## Results

**Fig. 1.**
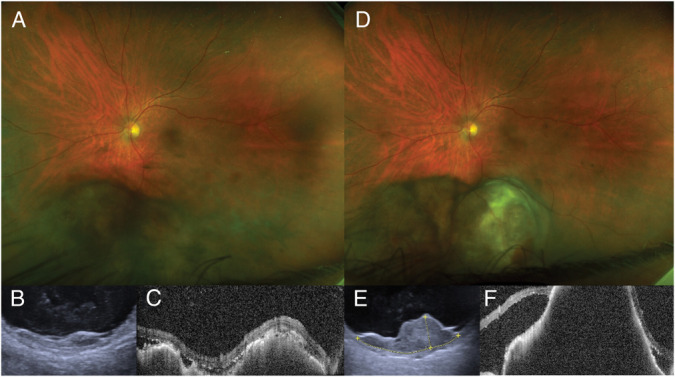
Clinical features of the intraocular tumor at the initial presentation (**A**–**C**) and 3 months later (**D**–**F**). Ultra-wide-field fundus photography shows an inferior mix-pigmented lesion with scattered retinal hemorrhage (**A**) that enlarged significantly in 3 months (**D**). Ultrasound sonography demonstrates a multilobed choroidal lesion (**B**) with rapid growth (**E**). Optical coherence tomography shows a lump-bumpy surface of the tumor at baseline (**C**) and increased thickness and subretinal fluid at three months (**F**).

A 64-year-old White man with treated prostate cancer presented with mild vitreous hemorrhage and a mixed-pigmented bilobed-choroidal equatorial tumor that showed heterogeneous echogenicity. The tumor measured 14.8 mm in diameter and 2.0 mm in height, which enlarged rapidly to 5.1 mm in 3 months (Figure 1). Systemic surveillance showed no evidence of metastatic disease. Clinical differential diagnosis included peripheral exudative hemorrhagic chorioretinopathy, metastatic deposit, and melanoma. A diagnostic transretinal biopsy without vitrectomy was done with a 23-gauge vitrector. Mild, self-limiting vitreous hemorrhage was noted immediately following the biopsy. The sclerotomies were sutured and triple freeze-thawed with cryotherapy. Analysis of the specimen revealed a melanoma, the final clinical stage being cT3a, stage IIB (American Joint Committee on Cancer, eighth edition).

**Fig. 2.**
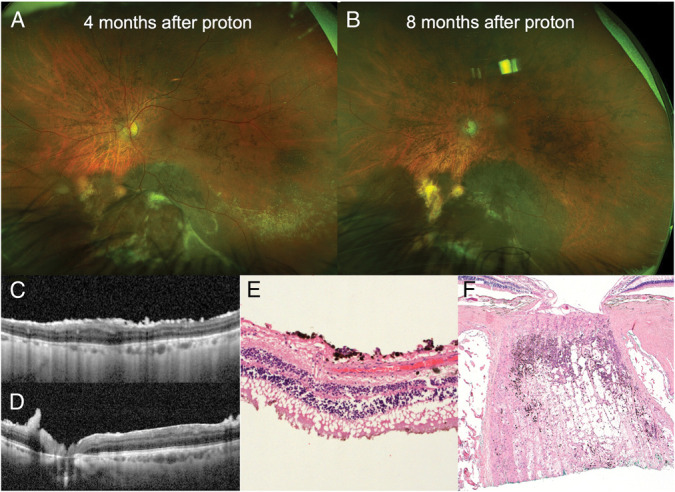
Fundus features and histopathology of a disseminated uveal melanoma following transvitreal diagnostic biopsy. **A** and **B.** Equatorial uveal melanoma is relatively flat after proton therapy but with surrounding flat extensions and increasing epiretinal pigmentation, which is clearly visible on optical coherence tomography (**C** and **D**). **E**. Histopathology confirms melanoma cells disperse on the retina (**E**) and infiltrate the cut end of the optic nerve (**F**).

**Fig. 3.**
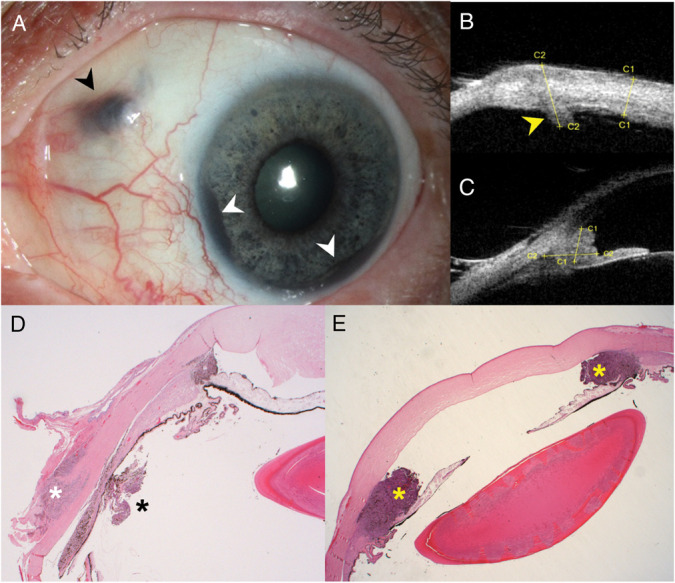
Clinical features and histopathology of a disseminated uveal melanoma following transvitreal diagnostic biopsy. **A.** Pigmented scleral mass at the prior vitrectomy scleral incision location (black arrowhead), which corresponds to the full-thickness scleral thickening on ultrasound biomicroscopy (**B**, yellow arrowhead). Scattered pigmentations on the iris and two back masses at the angle are also visible (**A**, white arrowheads). The angle lesion can be observed on ultrasound biomicroscopy (**C**). Histopathology confirms that the scleral mass in (**A**) contains melanoma cells infiltrating the sclera (white asterisk) and the pars plana (black asterisk). **E.** Ring melanoma is seen in the anterior chamber angle (yellow asterisks).

Immediate tumor treatment was precluded by persistent vitreous hemorrhage. A vitrectomy 5 weeks later cleared the blood and allowed tantalum markers insertion and then proton beam radiotherapy 10 weeks after the biopsy. The tumor height decreased to 3.4 mm eight months later, but liver metastasis developed at 12 months, and tebentafusp immunotherapy was initiated. At 14 months, increasing retinal pigmentation was noted across the fundus and iris (Figure 2), together with intraocular pressure surge and dark masses in the angle and 10 o'clock sclera (Figure 3). Owing to the suspicion of tumor dissemination, a secondary enucleation was performed. Pathology confirmed that the tumor disseminated across the retina, invaded the iris and formed ring melanoma, extended extrasclerally through the sclerotomy site, and infiltrated the optic nerve (Figures 2 and 3). The patient died of metastatic melanoma 2 years after the primary treatment.

## Discussion

Choroidal biopsy has a low tumor seeding rate if done with appropriate techniques and precautions.^1^ For transretinal biopsies, it is noteworthy that using 25-gauge needle, the scleral needle track showed incarcerated tumor cells in 6 of the 11 eyes (55%).^2^ Cryotherapy to the wound could possibly address these cells. In addition, by using vitrectomy trocars in needle biopsy, it was shown that there were no tumor cells found in the track.^3^ Nevertheless, in one of the 10 eyes, a clump of tumor cells are still observed in the anterior vitreous near the trocar.^3^ To prevent these cells to egress to the more vascularized subconjunctival space, adequate intraocular pressure control and suturing the sclerotomy wound should be considered.^1^ Moreover, using finer needles, such as 27-gauge or 30-gauge, has shown a very low complication rate.^4^ For transscleral biopsies, creating a lamellar scleral flap to cover the puncture site, using tissue glue to seal the wound, and immediate plaque radiotherapy after biopsy are proposed to minimize the risk of extraocular seeding.^1^

To the best of our knowledge, there are only 10 published cases of seeding following choroidal biopsy (Table 1).^5–11^ Nine of these cases had risk factors including multiple biopsies, additional intraocular surgeries, high-risk cytogenetics or cell morphology, or open biopsy. The present case had additional vitrectomy and a longer interval between biopsy and treatment which may increase the risk for tumor seeding. Unfortunately, cytogenetics analysis was unsuccessful. Nevertheless, the rapid growth of the tumor could imply the presence high-risk genetic mutations or chromosome aberrations.

**Table 1.** Published Cases of Tumor Seeding After Choroidal Biopsy

Author (year)	Biopsy Type	Biopsy Timing	Biopsy Technique	Baseline Hem	Post-Biopsy Hem	Additional Surgeries	High-Risk Genetics or Morphology	Definitive Treatment	Interval to Definitive Treatment	Seeding Location	Interval to Seeding
Caminal et al (2006)^8^	Dx	Pre-Tx	TS-FNAB (25 G)	—	Mild	—	—	Plaque	—	Epibulbar	8 mo
Raja et al (2011)^11^	Progx	Pre-Tx	TR-Vitrector (25 G)	—	—	—	M3	Proton	3 wk	Epibulbar	4 mo
Schefler et al (2013)^9^	Dx	Pre-Tx	Open biopsy	VH, Subretinal	—	PPV x2	—	Enucleation	—	Epibulbar and orbit	—
Schefler et al (2013)^9^	Dx	Pre-Tx	Two biopsies, technique unkown	—	—	—	—	Plaque	—	Eyelid and orbit	∼9 y
Schefler et al (2013)^9^	Progx	Pre-Tx	FNAB	—	—	PPV x2	—	Plaque	Immediate	Orbit	21 mo
Schefler et al (2013)^9^	—	Pre-Tx	FNAB	—	—	—	—	Not treated	—	Epibulbar	5 mo
Mashayekhi et al (2016)^10^	Progx	Pre-Tx	TS-FNAB (27 G)	Subretinal	No	—	M3	Plaque	Immediate	Epibulbar	18 mo
Koch et al (2017)^5^	Dx	Pre-Tx	TR-Vitrector (25 G)	—	—	—	M3	Plaque	—	Epibulbar and pars plana	24 mo
Ndulue et al (2020)^7^	—	Pre-Tx	TS-FNAB (25 G), partial thickness scleral incision, suture	—	Mild	—	Epithelioid	Plaque	—	Epibulbar, CB, AC	20 y
Mashayekhi et al (2021)^6^	Dx/Progx	Pre-Tx	TR-FNAB (27 G)/TS-FNAB (27 G), cryo, sutue	VH	Mild	—	M3	Plaque	2 wk	Epiretina, retina, optic nerve, central retinal vessels	14 mo
Present case (2025)	Dx	Pre-Tx	TR-Vitrector (23 G), cryo, suture	VH	Severe	PPV x1	—	Proton	10 wk	Epibulbar, optic nerve, AC, Epiretina, vitreous	14 mo

AC, anterior chamber; CB, ciliary body; cryo, cryotherapy; Dx, diagnostic; FNAB, fine-needle aspiration biopsy; G, gauge; Hem, hemorrhage; M3, monosomy 3; PPV, pars plana vitrectomy; Progx, prognostic; Tx, treatment; TS, transscleral; TR, transretinal; VH, vitreous hemorrhage.

The presence of vitreous hemorrhage both at baseline and after biopsy in the present case further posed concerns. Vitreous hemorrhage at presentation was reported in less than 3% of uveal melanoma.^12^ In contrast, in a review of 39 cases with uveal melanoma and vitreous seeding, three of them (7.6%) had vitreous hemorrhage.^13^ making one suspect that the vitreous hemorrhage could lead to spreading of the tumor cells. Furthermore, prolonged fundus-obscuring vitreous hemorrhage following biopsy will affect the subsequent brachytherapy or tantalum markers implantation for proton beam and postpone definitive treatment, as in the present case. This might have given time for more tumor cells to spread via the biopsy track to the other parts of the eye.

Several options could expedite the definitive treatment in eyes that have biopsy complicated by severe vitreous hemorrhage. Sequential or combined vitrectomy and plaque or markers insertion is a common approach.^14^ However, this means that the vitrectomy will be performed in an eye with an active and breached tumor mass and that the surgery may circulate the viable tumor cells in the eye, which significantly increases the risk of seeding. Alternatively, ultrasound-guided plaque insertion can be done when fundal view is poor. In addition, several groups are moving toward magnetic resonance imaging and computed tomography-guided proton therapy planning.^15^ Nevertheless, these approaches are technically challenging and await validation.

To further shorten the gap between biopsy and treatment, the definitive treatment can be prepared at the time of the biopsy. This is less of an issue for prognostic biopsies since the biopsy is usually together or even after the treatment.^1^ For diagnostic biopsies, one option is to request a rapid intraoperative pathology evaluation and start the treatment once the diagnosis is made. This will avoid unnecessary treatment but require the availability of pathology resources and expertise. The alternative method is to preplace the fiducial markers together with the diagnostic biopsy. Since the fiducial markers rarely cause complications, this option is worth considering. A more aggressive but safe alternative is to place the plaque immediately during the diagnostic biopsy. This is reasonable if the differential diagnosis is choroidal melanoma versus metastasis since both can be treated by brachytherapy, although the apex doses differ. However, if the pathology diagnosis reveals another benign diagnosis, such as peripheral exudative hemorrhagic chorioretinopathy, the placement of the plaque will be unnecessary; thus, this approach would need a thorough discussion with the patient beforehand. Notably, all the aforementioned approaches can only shorten the interval between biopsy and treatment, and the treatment only addresses the main tumor. If the cells were to spread together with the vitreous hemorrhage, the eye would still be at risk for tumor dissemination.

In conclusion, in this study, we report a case that had an atypical presentation of choroidal melanoma and underwent diagnostic biopsy. The persisting vitreous hemorrhage following the biopsy resulted in a 2.5-month gap between the biopsy and proton beam radiotherapy. Subsequently, dissemination of the melanoma was found. Several possible options are proposed to prevent this rare but serious and possibly serious complication.
